# Strukturwandel: Zukunftsangst in der Industrie

**DOI:** 10.1007/s10273-021-3007-1

**Published:** 2021-09-21

**Authors:** Marten von Werder, Ralf Rukwid

**Affiliations:** FB Grundsatzfragen und Gesellschaftspolitik, IG Metall Vorstand, Alte Jakobstraße 149, 10969 Berlin, Deutschland

**Keywords:** J28, L25, O33

## Abstract

Die Corona-Krise überlagert in der öffentlichen Wahrnehmung, wie stark der Strukturwandel in der Industrie bereits zu weitreichendem Arbeitsplatzabbau führt. Strukturwandel ist aber nicht gleich Strukturwandel: Die Art und Weise, wie Digitalisierung und Dekarbonisierung politisch umgesetzt werden, beeinflusst maßgeblich, wie sich die Aussichten für Beschäftigte in der Industrie entwickeln. Gerade kleine Betriebe scheinen unabhängig von ihrer Wirtschaftlichkeit gefährdet, da die Rahmenbedingungen der Transformation zu unsicher sind. Eine umfassende Beschäftigtenbefragung zeigt: Die Verunsicherung in den Leitbranchen der hiesigen Industrie ist groß. Die Beschäftigten fürchten vielerorts um ihre Jobs, hoffen auf stärkere Weiterbildung und eine gezielte aktive Industriepolitik — die nächste Bundesregierung ist hier gefordert.

## Abstract (en)

The coronavirus crisis obscures the extent to which structural change leads to widespread job losses in the German industrial sector. However, not all structural change is the same: the choice of policy approaches with which digitalisation and decarbonisation are implemented has significant influence on how the prospects for employees in this sector develop. Small businesses in particular seem to be at risk, regardless of their profitability, because the framework conditions for transformation are too uncertain. A comprehensive employee survey shows that there is great uncertainty in the leading sectors of the German industry. In many places, employees fear for their jobs and hope for more on-the-job training and a targeted, active industrial policy — this is a challenge for the next federal government.

Die IG Metall hat während der Corona-Krise zwei Befragungsformate durchgeführt: *Erstens* wurden in der „Corona-Lage-Erhebung“ (CL) wiederkehrend Betriebsrät:innen zur wirtschaftlichen und arbeitsschutzbezogenen Situation der Betriebe befragt.[1] *Zweitens* wurde im September 2020 eine Beschäftigtenbefragung (BB) in den von der IG Metall betreuten Betrieben via Online-Fragebogen durchgeführt.[2] Wir präsentieren einige der zentralen Ergebnisse dieser Umfragen und verknüpfen sie mit der Debatte um industriepolitische Richtungsentscheidungen.

1 Die CL-Erhebung erfolgte bisher fünfmal, erstmals im April 2020, zuletzt im Februar 2021. Es nehmen regelmäßig gut 5.000 Betriebe aus dem Organisationsbereich der IG Metall teil. Da im April 2020 zwei Befragungen stattfanden, enthält Abbildung 1 nur den zweiten April-Wert.

2 Der Datensatz zur Beschäftigtenbefragung umfasst vollständige Datensätze von 199.781 Beschäftigten aus 6.550 Betrieben. Die Befragung zielte auf die Arbeitssituation während der Corona-Pandemie mit einem besonderen Fokus auf der Betroffenheit durch Kurzarbeit sowie den Rahmenbedingungen für die Arbeit im Homeoffice. Die Daten können auf Anfrage in anonymisierter Form für wissenschaftliche Auswertungen zur Verfügung gestellt werden.

**Dr. Marten von Werder**, Referent beim Vorstand der IG Metall, Funktionsbereich Grundsatzfragen und Gesellschaftspolitik, Berlin.

**Dr. Ralf Rukwid**, Referent beim Vorstand der IG Metall, Funktionsbereich Grundsatzfragen und Gesellschaftspolitik, Berlin.

Insgesamt ist die Zusammensetzung der Industriebelegschaft durch öffentliche Daten nicht präzise erfasst. Allerdings gibt es verschiedene Datenquellen, die darauf hindeuten, dass die BB wesentliche Merkmale der Grundgesamtheit gut abbildet: Die Verdienststrukturerhebung (VSE)[3] des Statistischen Bundesamts weist z. B. aus, dass 19,9 % der Beschäftigten in der Metall- und Elektroindustrie (MuE) weiblich sind. Beschränkt man sich auf diesen MuE-Teil der BB liegt der Anteil von weiblichen Befragten bei 19,3 %. Laut VSE liegt das Durchschnittsalter in der MuE bei 44 Jahren, in den Daten der BB liegt das Durchschnittsalter leicht oberhalb der Altersgruppe von 40 bis 44 Jahren. Leichte Abweichungen ergeben sich bei der Branchenzusammensetzung: Im Vergleich zur VSE ist der Maschinenbau (27 % versus 20 %) in den BB-Daten unterrepräsentiert, während der Bereich zur Herstellung von Kraftwagen(-teilen) deutlich überrepräsentiert ist (23 % versus 43 %).[4] Außerdem zeigen sich Abweichungen in Bezug auf das Tätigkeitsfeld: Daten der Bundesagentur für Arbeit (BA)[5] weisen darauf hin, dass etwa 3/4 der Beschäftigten im Organisationsbereich der IG Metall in Produktions- oder produktionsnahen Berufen angestellt sind. Die Daten der BB unterschätzen diesen Anteil (51 %)[6]. In der Folge werden die Angaben deshalb im Regelfall auf

3 Die VSE ist letztmalig 2020 erschienen, mit Daten aus 2018.

4 Eine naheliegende Erklärung für beide Effekte ist die Betriebsgrößenstruktur: Im Maschinenbau sind die Betriebe oft kleiner, im Kraftwagenbau hingegen größer und gewerkschaftlich besser erschlossen.

5 Die Datenbasis stellt hier eine Sonderauswertung der Statistik der BA zu sozialversicherungspflichtig Beschäftigten nach Branchen (WZ 2008) und Berufshauptgruppen (KldB 2010) dar, zusammengefasst nach typischen Blue- und White-Collar-Berufssegmenten.

6 Dieses Muster ist bei Online-Befragungen typisch, da Beschäftigte in der Produktion – anders als Beschäftigte im indirekten Bereich – keinen Zugriff auf einen PC am Arbeitsplatz haben (Allmendinger und Schröder, 2021). Unter den besonderen Voraussetzungen der Corona-Krise wäre die Verzerrung sonst vermutlich aber noch deutlich größer, sodass die Beschäftigtenbefragung 2020 – anders als frühere Befragungen (z. B. IG Metall, 2017) – rein online durchgeführt wurde.

Abbildung 1
**Anteil der Betriebe, der Teile seiner Stammbelegschaft abbaut**

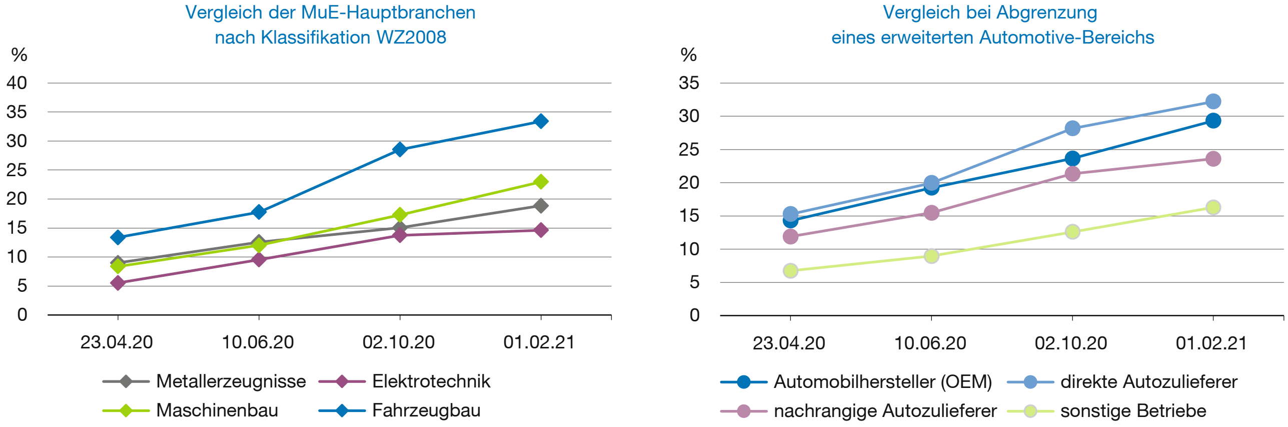

Quelle: Corona-Lage-Erhebung der IG Metall.

Branchen- und Tätigkeitsebene aufgeschlüsselt, um Verzerrungen durch die Gruppengrößen zu vermeiden.

## Wie die Corona-Krise die Bewältigung des Strukturwandels gefährdet

Der auf die Finanzkrise folgende wirtschaftliche Aufschwung ging mit einem deutlichen Anstieg der amtlichen Beschäftigtenzahlen in der hiesigen Industrie einher. Insbesondere der Maschinenbau (WZ 28) und der Kraftwagenbau (WZ 29) haben diesen Trend getragen. Schon vor der Corona-Krise setzte allerdings die Trendumkehr ein: Die Betriebe in der Industrie begannen Arbeitsplätze in Deutschland abzubauen. In den Organisationsbereichen der IG Metall sind zwischen 2019 und 2020 nach Jahresdaten des Statistischen Bundesamts etwa 116.000 Stellen abgebaut worden. Im Vergleich zum Mittel von 2020 sind es – ohne Kalenderbereinigung – im Juni 2021 weitere rund 100.000 Stellen. Durch die gegenwärtigen Lieferengpässe bei Halbleitern ist trotz guter Auftragslage und Nachholeffekten derzeit nur mit einer moderaten Relativierung dieser Zahlen im Jahresverlauf zu rechnen. Allerdings sollten diese konjunkturellen Aspekte ohnehin nicht den Blick auf strukturelle Entwicklungen verstellen: Abbildung 1 zeigt, dass der Anteil der Betriebe, der im Rahmen der CL-Erhebung angab, Beschäftigung abzubauen, in der zweiten Jahreshälfte 2020 verstärkt anstieg, als in der Industrie eigentlich längst ein konjunktureller Umschwung einsetzte.[7] Die Unterschiede zwischen den Branchen lassen sich nicht mithilfe der ebenfalls erhobenen Daten zur Insolvenzgefahr oder zur Auftragslage in der Corona-Krise erklären. Hinzu kommt: Von den Betrieben, bei denen Teile der Stammbelegschaft abgebaut wurden, haben 65 % von den umfassenden Zugangserleichterungen bei der Kurzarbeit zumindest zeitweise Gebrauch gemacht – und hätten dies natürlich auch weiterhin tun können. 38 % der Betriebe haben mindestens in einer Beobachtungsperiode der CL gleichzeitig Personal abgebaut und Kurzarbeit in Anspruch genommen.

Die Vermutung liegt deshalb nahe, dass der beobachtete Personalabbau zu einem Großteil eher strukturelle, denn konjunkturelle Gründe hat. Ein weiteres Indiz hierfür ist die exponierte Stellung des Fahrzeugbaus bzw. Automotive-Bereichs in Abbildung 1, der besonders von den Strukturumwälzungen der Dekarbonisierung erfasst wird. In der Literatur wurde die Gefahr für die Beschäftigung vielfach herausgearbeitet (Bauer et al., 2018; Helmrich et al., 2018; Hagedorn et al., 2019; Bernhard et al., 2020; Falck et al., 2021). Es sei zudem betont, dass sich der laufende Strukturwandel wesentlich von vorherigen Perioden dieser Art unterscheidet – und deshalb auch verbreitet unter dem Schlagwort „Transformation“ diskutiert wird.

Der gegenwärtige Strukturwandel wird unter anderem von der Dekarbonisierung und der Digitalisierung getrieben. Der disruptive Charakter beider Treiber geht mit einer so erhöhten Unsicherheit für Unternehmen einher, dass sie die Investitionsbereitschaft deutlich mindert (Bofinger, 2019). Die Wirtschafts- und Industriepolitik ist deshalb besonders gefordert. Ob ein Betrieb den Strukturwandel erfolgreich bewältigen kann, hängt nun wesentlich davon ab, welcher politische Rahmen für Dekarbonisierung und Digitalisierung gewählt wird. Beide Treiber gehen mit so wesentlichen infrastrukturellen und regulativen Anforde-

7 Die der Abbildung 1 zugrundeliegenden Betriebe bauten Teile ihrer Stammbelegschaft ab, also zusätzlich zum Abmelden von Leiharbeiter:innen und Werkvertragsnehmenden. Diese sind hier nicht berücksichtigt.

rungen einher, dass eine übergeordnete Abhängigkeit der Unternehmen – und damit der Beschäftigten – von staatlichem Handeln besteht. Zwar ist es die marktwirtschaftliche Aufgabe von Unternehmen, Geschäftsmodelle dynamisch an die sich verändernden Umstände anzupassen. Doch diese Umstände werden in diesem Strukturwandel stärker umwelt-, handels- und infrastrukturpolitisch geprägt als bisher und unterliegen deshalb stärker den Volatilitäten der politischen Auseinandersetzung – als Beispiel mag man die inhaltlich nachvollziehbare, aber in der Umsetzung hastige Verschärfung der $CO_2$-Reduktionsziele aus dem Frühjahr 2021 nehmen, die einen laufenden industriellen Strukturwandel noch einmal deutlich beschleunigt, ohne einen konkreten Umsetzungspfad und staatliche Unterstützungserfordernisse mitzudenken. Die durch die gebotene Pandemiebekämpfung verursachte konjunkturelle Krise hat diese Konstellation nur noch verschärft: Viele Betriebe haben in der Krise viel von der Liquidität verloren, die sie für die Investitionen in diesem Strukturwandel brauchen (vgl. als jüngstes Beispiel DIHK, 2021). Die Kehrseite dieser – im weiteren Sinne – industrie- und umweltpolitischen Regelungsbedarfe liegt in einer sozial-, arbeitsmarkt- und qualifizierungspolitischen Verantwortung der kommenden Bundesregierung.

## Personalabbau aus Sicht der Beschäftigten

In der BB sind auch 1.400 Beschäftigte zu Wort gekommen, die angaben, ihren Arbeitsplatz während der Corona-Krise verloren zu haben. Zunächst fallen hier die zu erwartenden Charakteristika auf: Leiharbeiter:innen, befristet Beschäftigte und durch Werkvertrag Beschäftigte sind in dieser Gruppe überrepräsentiert. Auch über 50 Jahre alt zu sein oder in Betrieben mit weniger als 1.000 Beschäftigten zu arbeiten, geht mit einem signifikant höheren Risiko einher, in der Krise den Job verloren zu haben. Andere Merkmale aber verwundern eher: Etwa 20 % derer, die ihren Arbeitsplatz verloren haben, waren in den Bereichen „Forschung und Entwicklung“ (FuE) oder „IT“ angestellt und nicht signifikant sicherer vor dem Arbeitsplatzverlust als z. B. Beschäftigte in der konjunkturell eher abhängigen Produktion – das gilt auch, wenn Branche, Betriebsgröße und Auftragslage berücksichtigt werden. 38 % hatten eine Hochschulausbildung oder waren Meister:innen/Techniker:innen/Fachwirt:innen – diese Gruppe war damit nicht signifikant weniger von Arbeitsplatzabbau betroffen, als Beschäftigte mit Ausbildung. 80 % gehörten zu Stammbelegschaften, d. h. ihre Anstellungsart trug keine Merkmale prekärer Beschäftigungsformen.

Die Ergebnisse zeigen, dass die Sicherheit eines Arbeitsplatzes nur sehr eingeschränkt durch Merkmale gewährleistet werden kann, die im Einflussbereich des Individuums liegen. Das in der öffentlichen Debatte vorherrschende Bild einer geringeren Strukturwandel-Betroffenheit von Beschäftigten mit hohem Bildungsniveau oder in bestimmten Tätigkeitsbereichen lässt sich mit Blick auf die aktuelle Arbeitsplatzsicherheit in der Corona-Krise nicht bestätigen.

Abbildung 2

**Beschäftigtenanteil, der Arbeitsplatz gefährdet sieht**

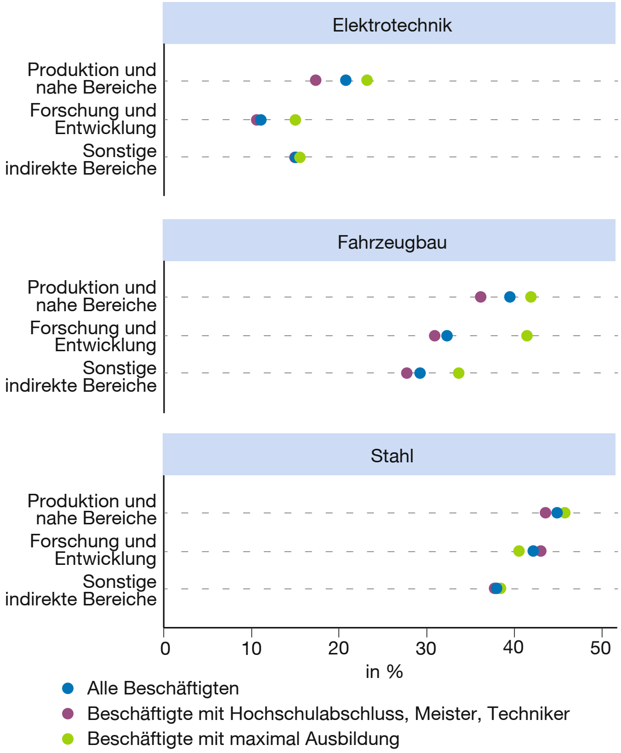

Quelle: Beschäftigtenbefragung 2020 der IG Metall.

In der direkten Befragung der Beschäftigten zeigt sich das Muster, dass es besondere Branchenspezifika gibt, welche die Arbeitsplatzsicherheit bestimmen. Abbildung 2 zeigt, wie der Anteil von Beschäftigten in ausgewählten Branchen, Tätigkeitsbereichen und für Qualifizierungsgrade variiert, die ihren eigenen Arbeitsplatz für gefährdet halten. Die Abbildung stellt dabei den Fahrzeugbau und den Stahlsektor heraus, zwei besonders vom Strukturwandel betroffene Branchen. In den Betrieben dieser Branchen zieht sich die Angst vor dem Arbeitsplatzverlust durch ganze Belegschaften. So sehen etwa 40 % der Beschäftigten im Fahrzeugbau und im Stahlsektor ihren eigenen Arbeitsplatz gefährdet. Bei knapp 796.000 Beschäftigen im Fahrzeugbau wären das mehr als 300.000 Beschäftigte. Und auch hier zeigt sich: Hochqualifizierte Beschäftigte im Tätigkeitsbereich FuE ähneln im Hinblick auf die Arbeitsplatzsicherheit ihren betriebs-/brancheninternen Kolleg:innen in der Produktion eher als den branchenfremden Beschäftigten desselben Tätigkeitsbereichs.

Nun ist die Sorge um den eigenen Arbeitsplatz in einer konjunkturellen Krise nichts Außergewöhnliches. Es ist deshalb wohl weniger das durchschnittliche Niveau der Gefährdung entscheidend als es die Unterschiede zwischen den Branchen sind. Das Niveau der subjektiven Gefährdung sollte gleichwohl verdeutlichen, zu welch grundlegender Verunsicherung der Strukturwandel bis tief in die Stammbelegschaften beschäftigungsreicher Branchen führt. Die Herausforderungen des Strukturwandels lassen sich am Beispiel des Fahrzeugbaus zeigen. Die IG Metall hat bereits Ende 2019 eine Umfrage unter Betriebsrät:innen und Betriebsbetreuer:innen der IG Metall gemacht, deren Betriebe zumindest einen Teil ihres Umsatzes mit Teilen für die KfZ-Herstellung machen.[8] Abbildung 3 zeigt den Anteil der Betriebe in diesem erweiterten Automotive-Bereich und die Zahl der Beschäftigten über die unterschiedlichen Grade der betrieblichen Abhängigkeit speziell vom Verbrennungsantrieb. Zumindest zum Ende 2019 machte der Verbrennungsantrieb in noch über 27 % der Betriebe mindestens 3/4 des Umsatzes aus. Betriebe mit dieser starken Abhängigkeit beschäftigten Ende 2019 etwa 440.000 Beschäftigte.[9]

Ende 2019 hatten noch nicht alle hiesigen Automobilhersteller (sogenannte OEM – Original Equipment Manufacturer) den Wechsel zur E-Mobilität offensiv kommuniziert. 2020 gab es jedoch eine verstärkte Positionierung in diese Richtung. Bekanntlich ist die Produktion von batterieelektrisch angetriebenen Autos weniger komplex und damit weniger arbeitsintensiv als die Produktion von „Verbrennern“. Außerdem entfällt ein großer Teil der Wertschöpfung auf die Batterie, diese werden allerdings noch überwiegend von außereuropäischen Produzierenden zugekauft. Batterieelektrische Mobilität geht deshalb – ceteris paribus – mit einem Beschäftigungsverlust in der Automobilindustrie einher (Bauer et al., 2018). Die OEM versuchen oft, dies durch eine Insourcing-Strategie zu kompensieren: Sie führen wieder Produktionsschritte selber durch, die zuvor bei Zulieferern lagen. Die Umorientierung in das batterieelektrische Zeitalter ist deshalb für viele Zulieferbetriebe noch schwieriger umzusetzen als für die OEM: Gerade kleinere Zulieferbetriebe stehen in einem strategischen Abhängigkeitsverhältnis von den OEM, die sie beliefern. Sie sind häufig auf die Herstellung weniger Komponenten und Teile spezialisiert und vielfach ist noch offen, inwieweit sich die Geschäftsmodelle in eine elektrifizierte Zukunft der Mobilität übertragen

8 Bei dieser Befragung wurden Informationen zu insgesamt 1.969 Betrieben gesammelt. Vornehmlich sind Betriebe aus dem Fahrzeugbau im Sinn der offiziellen Branchenklassifikation WZ2008 erfasst, aber auch Zulieferbetriebe aus anderen Branchen wie dem Maschinenbau. Bei 1.378 Betrieben hängt der Umsatz in relevantem Umfang von der Herstellung von Verbrennerfahrzeugen ab.

9 Die Beschäftigtenzahlen spiegeln die Summe der Gesamtbeschäftigtenzahlen all dieser Betriebe. Die Abbildung sähe etwas weniger drastisch aus, wenn nur die Zahl der Betriebe eingehen würde.

Abbildung 3

**Umsatzabhängigkeit vom Verbrennungsmotor im erweiterten Automotive-Bereich**

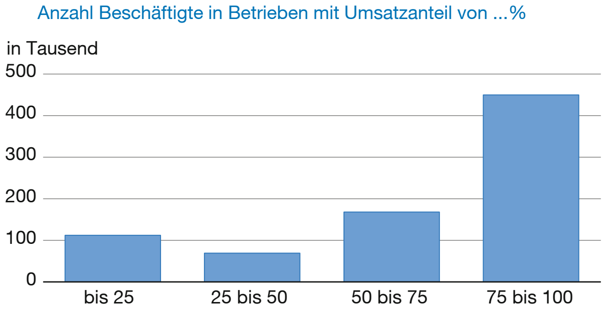

Quelle: Verbrennererhebung 2019 der IG Metall.

lassen. Die nun laufende Übergangszeit ist dabei mit Blick auf die Beschäftigtenzahlen besonders trügerisch, da im Grunde Doppelstrukturen bestehen – einerseits für die sich noch gut verkaufenden Verbrenner, andererseits für den anlaufenden Absatz von E-Autos. Ungewiss ist aber, wie lang diese Doppelstrukturen noch Beschäftigung in Deutschland halten. Hinzu treten die betrieblichen Herausforderungen der Digitalisierung: Absehbare Trends wie das autonome Fahren oder die Vernetzung des Verkehrs erhöhen den Anteil der digitalen Wertschöpfung am Auto und schieben das Geschäftsmodell der ganzen Branche zusehends in einen Bereich, in dem europäische Unternehmen abhängig von US-amerikanischen und asiatischen Digitalkonzernen sind und in dem neue Wettbewerber Vorteile besitzen.

Fragt man die Beschäftigten nach den Aussichten, bezweifeln viele, dass ihre Betriebe eine Strategie für die Transformation haben. Besonders deutlich wird der Zusammenhang nach Betriebsgröße: Im Fahrzeugbau geht nur knapp 1/3 der Beschäftigten in Betrieben mit maximal 300 Beschäftigten von einer Zukunftsstrategie ihrer Betriebe aus. Bei Großbetrieben mit mehr als 20.000 Beschäftigten sind dies fast 70 %. Viele Beschäftigte erleben die eigene Arbeitsplatzunsicherheit deshalb als ein strategisches Versagen ihrer Betriebe.

## Hebel gegen den Arbeitsplatzabbau

Die BB der IG Metall fragt neben den betriebs- und tarifpolitischen Erfordernissen auch eine Bandbreite von Einschätzungen der Beschäftigten dazu ab, wie mit dem Strukturwandel wirtschaftspolitisch umgegangen werden sollte. Während sich zwar Mehrheiten der Beschäftigten z. B. für höhere Steuern auf hohe Einkommen und Vermögen oder für einen Ausbau des Sozialstaats aussprechen, zeigt sich auch bei diesen Fragen eine gewisse Heterogenität im Antwortverhalten: Die Einschätzungen variieren über Tätigkeitsfelder, Ausbildungsniveaus oder Alter. An dieser Stelle

Abbildung 4
**Zustimmung zu Qualifizierungsmaßnahmen**

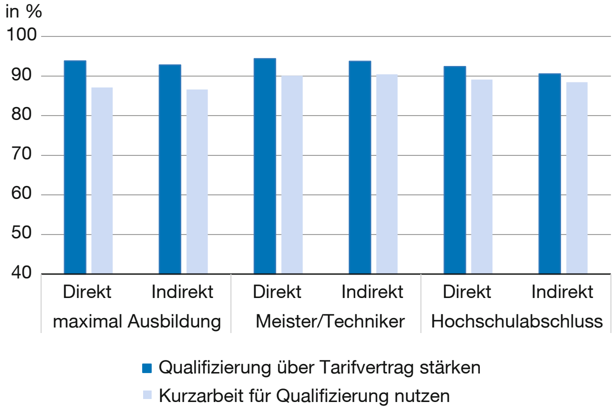

Quelle: Beschäftigtenbefragung 2020 der IG Metall.

soll deshalb speziell auf zwei politische Handlungsfelder aufmerksam gemacht werden, bei denen sich die Beschäftigten mit einer bemerkenswerten Einigkeit positionieren.

Abbildung 4 zeigt den Beschäftigtenanteil, der qualifizierungspolitischen Maßnahmen in der BB zugestimmt hat, aufgeschlüsselt nach Tätigkeitsbereich (direkt/indirekt)[10] und Ausbildungsniveau. Etwa 90 % der Beschäftigten stimmen der Forderung zu, die Qualifizierungsmöglichkeiten für Beschäftigte über tarifpolitische Angebote zu stärken. Die Zustimmung, Phasen in Kurzarbeit verstärkt für die Qualifizierung der Beschäftigten zu nutzen, ist im Niveau etwas geringer, aber insgesamt ebenso einhellig. Diese hohen Zustimmungswerte finden sich auch über alle Altersgruppen. Diese Daten sind ein deutliches Zeichen dafür, dass die Beschäftigten sich durchaus darüber bewusst sind, dass sich ein hoher Anteil qualifizieren muss, um seine Beschäftigungsperspektiven im Strukturwandel zu wahren. Die Gesetzgebung hat hier insofern reagiert, als dass mit dem Beschäftigungssicherungsgesetz neue Fördermöglichkeiten für Qualifizierungsaufwände in Phasen von Kurzarbeit geschaffen wurden – inwiefern diese Möglichkeiten aktuell und künftig auch praktische Anwendung finden, ist aber noch offen. Eher zeichnet sich ab, dass es weiterer Instrumente bedarf, um den Beschäftigten finanzielle und zeitliche Spielräume für Weiterbildung einzuräumen (vgl. den Vorschlag zum Transformations-Kurzarbeitergeld der IG Metall). Auch eine zusammen mit dem Institut der deutschen Wirtschaft erstellte Studie (ETL-Mittelstandskompass, 2021) deutet an, dass speziell mittelständische Firmen zu wenig in die Weiterbildung ihrer Beschäftigten investieren und so einen verstärkten Fachkräftemangel riskieren.

10 Tätigkeiten in Produktion und produktionsnahen Bereichen werden als „direkt“ verstanden, während klassische Angestelltentätigkeiten und Dienstleistungen den „indirekten“ Bereich ausmachen.

Die IG Metall hat in der gerade abgeschlossenen Tarifbewegung in der MuE einen Schwerpunkt auf Weiterbildung gesetzt: Zukunftstarifverträge sollen gerade solche Betriebe dazu drängen, sich den Herausforderungen der Transformation zu stellen, die bisher davor zurückgeschreckt sind. Auch die Möglichkeit, die betriebliche Arbeitszeit zur Beschäftigungssicherung auf vier Tage zu reduzieren, schafft zeitliche Spielräume für Weiterbildung und Qualifizierung. Um diese Möglichkeiten tatsächlich nutzen zu können, braucht es allerdings bei vielen Betrieben eine noch klarere Vorstellung davon, wie sich ihre Geschäftsmodelle – und damit natürlich auch die Anforderungsprofile ihrer Beschäftigten – verändern.

Das zweite politische Handlungsfeld, das Beschäftigte in überragender Einhelligkeit sehen, ist das der aktiven Industriepolitik – ein Bereich also, der für viele Betriebe die Voraussetzungen und Rahmenbedingungen schaffen muss, um eine Weiterentwicklung des Geschäftsmodells zu ermöglichen. Abbildung 5 zeigt, inwiefern Beschäftigte aus unterschiedlichen Branchen, dargestellt über Qualifizierungsgrade und Tätigkeitsbereiche, der Aussage zustimmen, dass „Zukunftsfelder (Wasserstoffwirtschaft, künstliche Intelligenz, Batteriezellenfertigung…) durch aktive Industriepolitik gefördert werden müssen“. Auch hier zeigen sich nur geringe Abweichungen über Qualifizierungsgrade, Branchenzugehörigkeit oder Tätigkeitsbereiche. Die Zustimmung zu einer aktiven industriepolitischen Förderung von Zukunftsfeldern liegt durchgängig bei mindestens 80 % der Beschäftigten.

Die hohe Zustimmung der Beschäftigten lässt sich auch als Zustimmung zu Maßnahmen wie der Wasserstoffstrategie der Bundesregierung oder den im Konjunkturpaket 2020 angelegten Maßnahmen zur Batteriezellforschung interpretieren – allerdings fehlt es bei diesen Vorhaben oft noch an der konkreten Umsetzung. Andere industriepolitische Bereiche liegen eher brach: z. B. punktuelle öffentliche Beteiligungen, um KMU bei der Transformation ihrer Geschäftsmodelle zu unterstützen oder die gegenwärtige Förderung von Investitionen, die erst mit mittelfristig realistischen $CO_2$-Preisen betriebswirtschaftlich rentabel wären. Dies gilt z. B. für den Stahlbereich, in dem die Investitionszyklen sehr lang sind und wo ein Ausgleich durch Differenzverträge erfolgen könnte. Ebenso zeigt die Zustimmung, dass die bisweilen verkopften Debatten um die ordnungspolitische Legitimität staatlicher Fördermaßnahmen an der Stimmung unter den Beschäftigten vorbeigehen. Man mag darauf erwidern, dass die Beschäftigten nicht die Expertise hätten, die wirtschaftspolitischen Prozesse hinter dem Aufbau neuer Industriezweige zu beurteilen. Aus dreierlei Gründen ist das allerdings nicht stimmig: *Erstens* haben gerade die Fachkräfte in den Betrieben oft einen sehr guten Einblick in die Lücken zwischen technischen Möglichkeiten und

Abbildung 5

**Zustimmung zu „Zukunftsfelder müssen durch aktive Industriepolitik gefördert werden“**

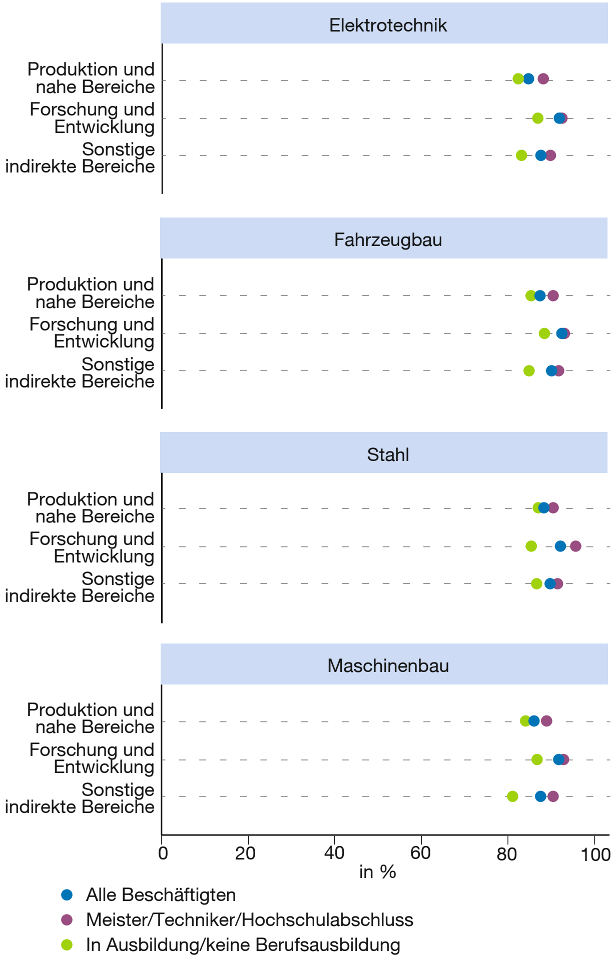

Quelle: Beschäftigtenbefragung 2020 der IG Metall.

betriebswirtschaftlichen Rahmenbedingungen, aus denen sich der industriepolitische Handlungsbedarf zumindest zum Teil ableitet. *Zweitens* kommen zahlreiche Expert:innen mit Verweis auf den disruptiven Charakter von Digitalisierung und Dekarbonisierung und der damit einhergehenden großen Unsicherheit für private Investor:innen ebenfalls zu der Empfehlung, neben einer horizontalen Industriepolitik auch eine differenzierende Industriepolitik zu etablieren (Bardt, 2019; Gerlach und Ziegler, 2019). *Drittens* sollten die Sorgen der Industriebeschäftigten in Deutschland auch mit Blick auf die kommende Bundestagswahl ernstgenommen werden. Die öffentliche Debatte schien hier – z. B. mit dem ursprünglichen Vorschlag zur nationalen Industriestrategie – bereits weiter zu sein, als sie es derzeit ist.

**Title:** ***Structural Change: Industry's Fear of the Future***

**JEL Classification:** *J28, L25, O33*
